# c-Myc activates *BRCA1 *gene expression through distal promoter elements in breast cancer cells

**DOI:** 10.1186/1471-2407-11-246

**Published:** 2011-06-13

**Authors:** Yinghua Chen, Jinhua Xu, Stanley Borowicz, Cindy Collins, Dezheng Huo, Olufunmilayo I Olopade

**Affiliations:** 1 Center for Clinical Cancer Genetics and Global Health, Department of Medicine, University of Chicago, Chicago, IL, USA; 2 College of Medicine, University of Illinois at Chicago, Chicago, IL, USA; 3 Department of Health Studies, University of Chicago, Chicago, IL, USA

## Abstract

**Background:**

The *BRCA1 *gene plays an important role in the maintenance of genomic stability. BRCA1 inactivation contributes to breast cancer tumorigenesis. An increasing number of transcription factors have been shown to regulate *BRCA1 *expression. c-Myc can act as a transcriptional activator, regulating up to 15% of all genes in the human genome and results from a high throughput screen suggest that *BRCA1 *is one of its targets. In this report, we used cultured breast cancer cells to examine the mechanisms of transcriptional activation of *BRCA1 *by c-Myc.

**Methods:**

c-Myc was depleted using c-Myc-specific siRNAs in cultured breast cancer cells. *BRCA1 *mRNA expression and BRCA1 protein expression were determined by quantitative RT-PCR and western blot, respectively and *BRCA1 *promoter activities were examined under these conditions. DNA sequence analysis was conducted to search for high similarity to E boxes in the *BRCA1 *promoter region. The association of c-Myc with the *BRCA1 *promoter *in vivo *was tested by a chromatin immunoprecipitation assay. We investigated the function of the c-Myc binding site in the *BRCA1 *promoter region by a promoter assay with nucleotide substitutions in the putative E boxes. BRCA1-dependent DNA repair activities were measured by a GFP-reporter assay.

**Results:**

Depletion of c-Myc was found to be correlated with reduced expression levels of *BRCA1 *mRNA and BRCA1 protein. Depletion of c-Myc decreased *BRCA1 *promoter activity, while ectopically expressed c-Myc increased *BRCA1 *promoter activity. In the distal *BRCA1 *promoter, DNA sequence analysis revealed two tandem clusters with high similarity, and each cluster contained a possible c-Myc binding site. c-Myc bound to these regions *in vivo*. Nucleotide substitutions in the c-Myc binding sites in these regions abrogated c-Myc-dependent promoter activation. Furthermore, breast cancer cells with reduced BRCA1 expression due to depletion of c-Myc exhibited impaired DNA repair activity.

**Conclusions:**

The distal *BRCA1 *promoter region is associated with c-Myc and contributes to *BRCA1 *gene activation.

## Background

The human breast cancer susceptibility gene 1 product, BRCA1 is involved in important cellular processes, including DNA repair, and loss of BRCA1 can result in genomic instability. Loss of BRCA1 expression occurs in a subset of breast cancer cases, and inherited mutations of the *BRCA1 *gene account for about 5% of all breast cancer cases [1-8]. The regulation of *BRCA1 *expression has been studied extensively, including investigations of alternative mechanisms for reduced expression of *BRCA1 *in sporadic cases [9,10]. A set of transcription factors and/or co-factors has been shown to regulate *BRCA1 *expression through a region in close proximity to the *BRCA1 *transcription start site. This proximal promoter region of *BRCA1 *is bidirectional and includes a 218 bp intergenic region between the *BRCA1 *and *NBR2 *genes [11]. Within this region, it has been demonstrated that a short segment (-204 to -148) relative to *BRCA1 *exon 1a start site may be responsible for *BRCA1 *promoter activities [12]. Another report confirmed these findings using a different strategy of deletion analysis and showed that a slightly shorter region (-202 to -166) was required for *BRCA1 *promoter activation [13,14]. Further studies of this region (-202 to -166) revealed two sub-elements: 1) a RIBS element (-204 to -182) that binds to and is activated by a GABPα/β complex [15] and 2) a CREB/ATF1 element (-174 to -167) that acts as a constitutive transcriptional activation element bound by CREB [16,17]. In addition, the E2F family of transcription factors binds to two regions, -41 to -31 and -21 to -11, and activates or represses *BRCA1 *expression depending on the co-factors recruited [18,19], BRCA1 itself has been shown to be one of the co-factors [20]. An element (-40 to -25) that overlaps one of the E2F binding sites can be bound and activated by 53BP1 in a sequence-specific manner and functions as a positive regulatory element [21]. An ER-α transcription complex binds an AP1 element (+246 to +250) and activates *BRCA1 *transcription upon estrogen stimulation [22]. This ER-α dependent activation can be modulated by an aromatic hydrocarbon receptor complex that binds two consecutive xenobiotic-responsive elements located upstream of the ER-α binding region (+17 to +21 and +175 to +179) [23]. A relatively long segment in a 5 kb region in *BRCA1 *intron 2 that is highly conserved in multiple species contains a CNS-1 (Conserved Nucleotide Site-1) and CNS-2, which appear to act as repression and activation elements, respectively [24].

c-Myc is a transcription factor involved in growth, proliferation, differentiation, and apoptosis of cells and regulates up to 15% of human genes [25]. c-Myc regulates transcription through several mechanisms, and *cis*-regulatory elements modulate specific subsets of c-Myc targets. One of the *cis*-regulatory elements, E box, is common in c-Myc targeted genes [26]. Serial analysis of gene expression performed after adenoviral expression of c-Myc in primary human umbilical vein endothelial cells has implicated *BRCA1 *as one of the activated gene targets for c-Myc [27]. However, it was not clear whether c-Myc could transcriptionally regulate *BRCA1 *expression through a *cis*-regulatory element, particularly in breast cancer cells. In this report, we show that depletion of c-Myc is correlated with a reduction in *BRCA1 *mRNA and BRCA1 protein levels and decreased *BRCA1 *promoter activities were observed in the cells following depletion of c-Myc. On the other hand, ectopic expression of c-Myc activated *BRCA1 *promoter activities. DNA sequence analysis revealed two novel E boxes within the distal *BRCA1 *promoter. A chromatin immunoprecipitation assay demonstrated that c-Myc binds to these E boxes *in vivo*. Furthermore, we show that *BRCA1 *promoter/reporters containing nucleotide substitutions in these E boxes abrogate their c-Myc dependent activation. These data suggest that c-Myc activates *BRCA1 *expression through the E boxes in the distal *BRCA1 *promoter region. Finally we observed that cells treated with c-Myc specific siRNAs had reduced DNA repair activity, a biological process associated with BRCA1.

## Methods

### Cell culture

Breast cancer cells were obtained from the American Type Culture Collection (ATCC, Manassas, VA). MCF-7 cells (ATCC: HTB-22) were cultured in DMEM (Mediatech, Inc., Manassas, VA) supplemented with 10% fetal bovine serum (Lonza, Inc, Allendale, NJ) and 1% antibiotic-antimycotic solution (Mediatech Inc.). MDA-MB-231 cells (ATCC: HTB-26) were cultured in RPMI1640 (Invitrogen, Carlsbad, CA) supplemented with 10% fetal bovine serum (Lonza, Inc.) and 1% antibiotic-antimycotic solution (Mediatech, Inc.).

### Plasmid construction

A vector containing human *c-Myc *cDNA was obtained from ATCC (ATCC: 5233860) and used to construct a c-Myc-GFP expression vector designated pMYC-GFP. The full length c-Myc protein coding DNA fragment was inserted in the 5' end of *GFP *under the control of the cytomegalovirus (CMV) promoter. First the full-length coding region of *c-Myc *was amplified by PCR using primers (5'-TCCCGCGACG ATGCCCCTCA ACGTTAGCTTCA-3' and 5'-CACAAGAGTT CCGTAGCTGT TCAAGTTTGTG-3') and the *c-Myc *cDNA vector (ATCC 5233860) as a template. Then the PCR products were cloned into pcDNA3.1/CT-GFP-TOPO (Invitrogen) following the manufacturer's protocol, yielding pMYC-GFP. The c-Myc encoding sequence was confirmed by DNA sequence analysis to be identical to that of GenBank: BC000917.

A promoter/luciferase reporter construct containing the *BRCA1 *promoter region (-1714 to +42) in pGL4.10 (Promega, Madison, WI) was generated as follows. The required *BRCA1 *promoter region was amplified by PCR using the primer pair: 5'-CTA*GGTACC *TTGGGAGGGG GCTCGGGCAT GGC-3' and 5'-CAT*AAGCTT *CCAGGAAGTC TCAGCGAGCT CACG-3' (KpnI and HindIII sites were underlined, respectively) and human placental genomic DNA (Sigma-Aldrich, St. Louis, MO) was used as a template. The PCR products were digested with KpnI and HindIII, and the resulting fragments were inserted into pGL4.10 at identical sites. The resulting construct was named pCYL42. The inserted DNA sequence of the *BRCA1 *promoter was determined to be identical to that of GenBank: L78833 by DNA sequence analysis.

We used a QuikChange Site-Directed Mutagenesis Kit (Stratagene, La Jolla, CA) to generate E box nucleotide substitutions following the manufacturer's protocol. To generate single E box nucleotide substitution constructs, we used pCYL42 as the original template and corresponding E box mX and E box mY primer pairs. The primers (sense strands shown) used are shown below with nucleotide substitutions underlined: E box mX: 5'-AGATTGGCTC TTACAAAATG TCCCTCAAAA CGAC-3'; and E box mY: 5'-GCGAGGGCTG CTAGAAAATT GTCACCTCGC ATTCT-3'. To generate the double E box mutant, the mutagenesis reactions were conducted using the primers E box mX and E box Y nucleotide substitution constructs as templates. All constructs were confirmed by DNA sequence analysis.

The pMyc-TA-Luc and pTA-Luc plasmids used as c-Myc activated promoter/luciferase controls were purchased (Clontech Laboratories, Inc., Mountain View, CA). The pMyc-TA-Luc vector is a derivative of pTA-Luc and contains six tandem copies of the E box consensus sequence (CACGTG) located upstream of the minimal TA promoter, followed by the firefly luciferase gene.

### Small interfering RNA (siRNA)

All related reagents, including *c-Myc *siRNAs, control siRNA, and transfection reagents, were purchased from Qiagen (Valencia, CA). Transfections were performed according to the manufacturer's protocol.

### Quantitative reverse transcriptase PCR

Total RNA was extracted from cultured cells using the RNeasy mini kit (Qiagen). Reverse transcription reactions were performed with the SuperScript III First-Strand Synthesis System (Invitrogen) using 1 μg of DNase-treated RNA and oligo (dT) primer. Real-time PCR was performed in a Light Cycler (Roche Diagnostics, Indianapolis, IN) using the LightCycler FastStart DNA Master PLUS SYBR Green I kit (Roche Diagnostics). Primers for *BRCA1 *and glyceraldehyde-3-phosphate dehydrogenase (*GAPDH*) were used as previously described [9,28]. Thermal cycling for amplification of *BRCA1 *or *GAPDH *was initiated by heating at 95°C for 10 min, followed by 40 cycles of denaturation at 94°C for 10 sec, annealing for 10 sec at 54°C and 57°C for *BRCA1 *and *GAPDH*, respectively, and elongation at 72°C for 15 sec. After completion of the PCR cycles, melting curve analyses and electrophoreses of the products on 2% agarose gels were performed to validate generation of each specific, expected PCR product. The fold change in *BRCA1 *cDNA (target gene) relative to the *GAPDH *control was determined by the 2^-ΔCt ^method [29]. Experiments were conducted independently twice in triplicate.

### Western blot

Western blotting was carried out as previously described [30]. Mouse anti-BRCA1 antibody (AB-4, Oncogene, San Diego, CA), mouse anti-c-Myc antibody (sc-40, Santa Cruz Biotechnology, Inc., Santa Cruz, CA), and goat anti-actin antibody (sc-1616, Santa Cruz Biotechnology, Inc.) were used for western blotting. An ECL western blotting detection reagent kit (GE Healthcare, Buckinghamshire, UK) was used for detection.

### Transient transfection and luciferase assay

One day prior to transfection, 6 × 10^5 ^cells were seeded into one well of a six-well tissue-culture plate (BD Biosciences, San Jose, CA). After 24 hours, cotransfections were conducted. The transfection mixture contained a promoter/luciferase reporter construct (980 ng), a CMV/renilla luciferase vector, pGL4.75 (20 ng, Promega) as a transfection efficiency control, and an expression construct for c-Myc (pMYC-GFP, 1000 ng or other indicated amounts) or additional control vector pcDNA3.1/CT-GFP (Invitrogen) to give consistent amounts of total vector of 2000 ng. Then 6 μl of FuGENE HD (Roche Diagnostics) was added to the mixture, and cotransfections were done according to the manufacturer's instruction. Forty-eight hours after transfection, cell lysates were prepared using passive lysis buffer (Promega). Luciferase activities in the lysates were measured using a dual luciferase assay system (Promega) with a 1450 microbeta trilux jet scintillation and luminescence counter (PerkinElmer Life and Analytical Sciences, Downers Grove, IL). Experiments were performed in triplicate.

In reporter assays with depletion of c-Myc in MCF-7 and MDA-MB-231 cells, 24 hours after siRNA treatment, cotransfections were done using the mixture of pCYL42 (1000 ng) and pGL4.75 (20 ng) combined with 2 μl of FuGENE HD as described above. Luciferase activity was determined 48 hours after the transfection. Experiments were performed in triplicate.

### Chromatin immunoprecipitation

Chromatin immunoprecipitation (ChIP) assays were performed following the manufacturer's protocol (Upstate Biotechnology, Lake Placid, NY) using 2 × 10^6 ^MCF-7 or MDA-MB-231 cells. An anti-c-Myc antibody (sc-764, Santa Cruz Biotechnology, Inc.) or an anti-CTCF antibody (sc-15914, Santa Cruz Biotechnology, Inc) was used to precipitate DNA-protein complexes, and the normal isotype-matched IgG from the same species was used as negative control. The PCR primers used for detecting DNA fragments containing E boxes are: E box X: ChIPX-FW: 5'-AATGCAAAGA CCGTCCGCTG CCA-3'and ChIPX-RV: 5'-TCCACCCCTC AGCCCCAGTG TTT-3'; E box Y: ChIPY-FW: 5'-TGAAGGGCTC CTCCAGCACG-3' and ChIPY-RV: 5'-TGAGGGACCG AGTGGGCGAA-3', and non E box: ChIPNE-FW: 5'-CGAGAGACGC TTGGCTCTTT CTGT-3' and ChIPNE-RV: 5'-GCCCAGTTAT CTGAGAAACC CCAC-3'. The amplified PCR product size was 233 bps for the detection of E box X, 146 bps for E box Y, and 214 bps for non E box, respectively. The band densities of the targeted PCR products were quantified with the software QUANTITY ONE version 4.5.1 (Bio-Rad, Hercules, CA).

### DNA repair assay

We used a fluorescence-based DR-GFP reporter system with modifications [31,32]. The efficiency of homologous recombination was assessed using a restriction endonuclease I-SceI expression plasmid pCβASce and pDR-GFP, an I-SceI repair reporter plasmid composed of two differentially mutated GFP genes, one of which contained a unique I-SceI restriction site. A double strand break of DR-GFP plasmids created by I-SceI digestion was repaired by gene conversion to produce a functional GFP. The cells expressing GFP, representing homologous recombination activity, were detected with flow cytometry. Briefly, MDA-MB-231 cells were seeded in a well of a six well plate. On the next day, the cells were treated with equal amounts of siRNA against c-Myc or control siRNA with Hiperfect as instructed by the manufacturer (Qiagen). 48 hours later, pDR-GFP plasmid, along with either pCβASce (expressing endonuclease I-SceI to create double strand breaks (DSBs)) or pCAGGS (empty vector) were co-transfected into cells by Fugene HD (Roche) as described above. After incubation for 48 hours, cells were trypsinized, harvested, and finally suspended in PBS. Consequently, the cells were analyzed at the flow cytometry facility of the University of Chicago with a Becton Dickinson FCAScan (BD Biosciences). The channel FL-1 (green) and FL-2 (orange) were recorded and used to calculate the frequency of GFP positive cells.

### Statistical analysis

For quantitative RT-PCR, after logarithmic transformation, *BRCA1 *mRNA level was analyzed using ANOVA. Then the regression coefficients were transformed back to obtain the fold change or geometric mean difference between study groups [33].

Means and standard deviations (SDs) were calculated for relative luciferase activities, densitometry ratios, and homologous recombinant efficiencies. Two-tailed Student's *t*-tests were used to compare only 2 groups. ANOVA were implemented for comparisons with multiple groups. After the overall analysis, Tukey tests were used to make the pairwise comparison.

## Results

### The region -1714 to +42 of the BRCA1 promoter is sufficient for response to c-Myc activation

**Figure 1:**
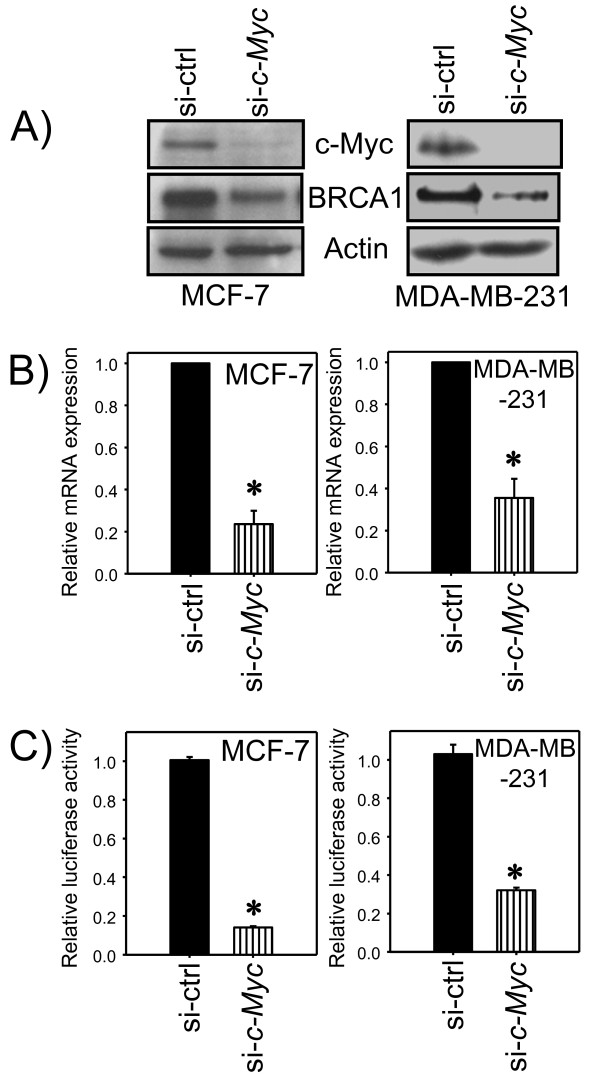
**Reduction of expressions of *BRCA1 *mRNA and BRCA1 protein, and *BRCA1 *promoter activity by depletion of c-Myc in breast cancer cells**. (A) MCF-7 or MDA-MB-231 cells were treated with *c-Myc *siRNA to deplete c-Myc expression, and total cellular c-Myc and BRCA1 protein levels were determined by western blot with actin levels serving as a protein loading control. (B) *BRCA1 *mRNA expression levels from cells treated with *c-Myc *siRNA were determined by quantitative RT-PCR. Real-time PCR was performed in two independent experiments in triplicate. Bars show mean ± SE. *: p < 0.01. (C) The *BRCA1 *promoter reporter vector pCYL42 was transfected into cells with depleted c-Myc by *c-Myc *siRNA treatments, and *BRCA1 *promoter activities were recorded. The data were presented as means of control siRNA as 1, bars show mean ± SD. *: p < 0.01.

**Figure 2:**
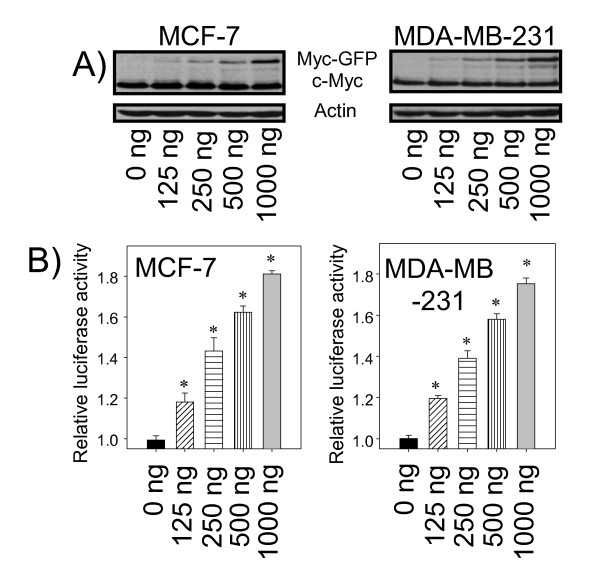
**Activation of *BRCA1 *expression by c-Myc in breast cancer cells**. A vector of overexpression *c-Myc *(pMyc-GFP) and the reporter/luciferase constructs were co-transfected into MCF-7 or MDA-MB-231 cells as describer in methods. The amounts of Myc-GFP expression vectors are as indicated. (A) The constitutively expressed Myc-GFP fusion protein (Myc-GFP) and endogenous c-Myc (c-Myc) from the same amounts of whole cellular protein extracts from each cell line are detected by western blot. Actins are used as the loading control (Actin). (B) *BRCA1 *promoter activities from whole cell lysates were measured. The luciferase data were normalized to the empty vector and compare to that without overexpression of c-Myc as 1 (column: 0 ng). Bars show mean ± SD. *: p < 0.01 as each indicated column compared to the 0 ng column.

*BRCA1 *has been implicated as a c-Myc target in a high throughput study using human umbilical vein endothelial cells overexpressing c-Myc [27]. Whether c-Myc can directly regulate *BRCA1 *expression at the transcriptional level is not clear. We successfully depleted c-Myc expression in the breast cancer cell lines MCF-7 and MDA-MB-231 through treatment with siRNA against the *c-Myc *gene. In both cell lines, transient transfection of *c-Myc *siRNA efficiently reduced c-Myc protein expression (Figure 1A). Correspondingly, BRCA1 protein levels were reduced upon depletion of c-Myc in both cell lines (Figure 1A). More importantly, *BRCA1 *mRNA levels in cells with depleted c-Myc protein were reduced to 24% in MCF-7 and 36% in MDA-MB-231 compared to the control siRNA treated cells (Figure 1B). To establish that *BRCA1 *expression was directly modulated by c-Myc through DNA regulatory elements in the *BRCA1 *promoter region, we constructed a *BRCA1 *promoter/luciferase reporter vector containing the *BRCA1 *promoter region -1714 to +42. *BRCA1 *promoter activity following depletion of c-Myc was significantly reduced compared to control in both cell lines (Figure 1C). These data suggest that reduced c-Myc might decrease *BRCA1 *expression in these two breast cancer cell lines through the *BRCA1 *promoter. To test whether this *BRCA1 *promoter region sufficiently responded to c-Myc activation, we monitored *BRCA1 *promoter activity in the presence of ectopically expressed functional c-Myc from transient transfections of pMYC-GFP into MCF-7 or MDA-MB-231 cells. We observed increasing expression of the Myc-GFP with increasing amounts of Myc-GFP expression vector used, while the endogenous c-Myc expression remained consistent (Figure 2A). In both cultured cell types, Myc-GFP protein levels produced at the maximal dose of the Myc-GFP expression vector were similar to the levels of endogenous c-Myc at the time when cells were used for luciferase assays (Figure 2A). In the presence of constitutively expressed Myc-GFP, *BRCA1 *promoter activity increased proportionally to increasing amounts of pMyc-GFP (Figure 2B). Taken together, these data indicated that c-Myc activates *BRCA1 *expression through the region of the *BRCA1 *promoter (-1714 to +42), which could contain c-Myc-mediated DNA regulatory elements.

### Putative E boxes in the -1714 to +42 bp region of the BRCA1 promoter

**Figure 3:**
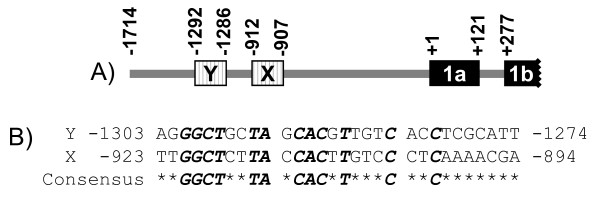
**A diagram of *BRCA1 *promoter region and the alignment of regions spanning putative E boxes in the *BRCA1 *distal promoter**. (A) The diagram is an illustration only representing E boxes in *BRCA1 *gene distal promoter and is not to scale. Positions of putative E boxes are marked by boxes with vertical lines, and regions of *BRCA1 *exon 1a and 1b (partial) are marked by filled boxes. The locations are relative to the transcription start site of exon 1a as +1. (B) Nucleotides that are conserved between two of the E boxes (bold and italic) are shown as the deduced consensus sequence listed below, while variable nucleotides are marked with asterisks: *.

c-Myc regulatory sites within gene promoter regions, such as the cognate hexanucleotide E box (CACGTG) and non-cognate E boxes have been well documented, but the DNA sequences of c-Myc binding regions could be more degenerate [26]. In order to identify c-Myc interacting elements, we used these E box sequences to search for possible matches in the -1714 to +42 region of the *BRCA1 *promoter, which is responsive to c-Myc activation. Two nucleotide regions, -1292 to -1286 (E box Y) and -912 to -907 (E box X), were found to be either identical to or with high similarity to E box sequences (Figure 3A). One nucleotide at position 4 of E box X; (-909: G to T) differed from the cognate E box sequence CACGTG, while seven nucleotides of E box Y were identical to the non-cognate E box sequence CACGTTG. An alignment of the putative E boxes and their adjacent regions is shown in Figure 3B. Almost half of the nucleotides (12 to 30) were identical in two DNA clusters, which indicated that these regions could serve as a tandem array that facilitates c-Myc-containing transcription factor binding (Figure 3B).

### c-Myc forms complexes with E boxes of the BRCA1 promoter *in vivo*

**Figure 4:**
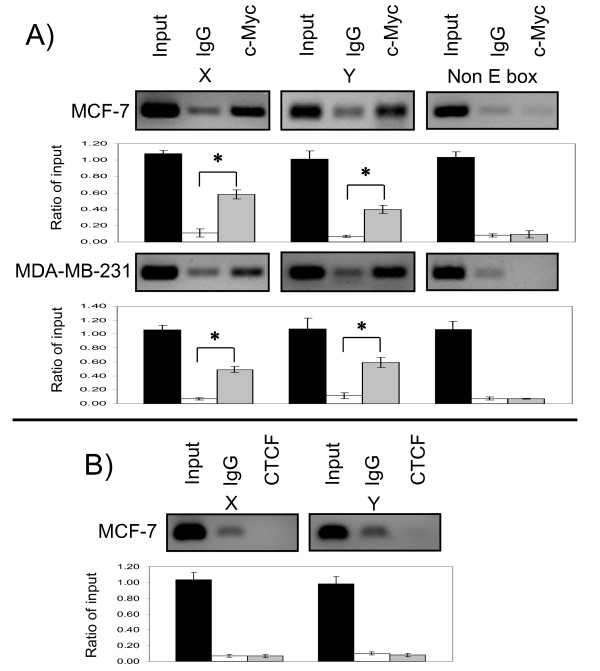
**c-Myc associates with the *BRCA1 *promoter regions containing E boxes *in vivo***. (A) Cross-linked protein-DNA complexes with formaldehyde were incubated with an anti-c-Myc antibody or control IgG. After immunoprecipitation, DNA is recovered from the protein-DNA complex, and the DNA fragments were subjected to PCR with a primer pair specific for detecting the distal *BRCA1 *promoter regions containing E box X, or E box Y, and indicated as panel X, or panel Y, respectively. Detection of a random region in *BRCA1 *promoter was shown as non E box panel. (B) An anti-CTCF antibody or control IgG were used for immunoprecipitation of CTCF bound protein-DNA complex, and the DNA fragments recovered were subjected to PCR for detecting regions containing E box X, or E box Y. An aliquot (1%) of total DNA without immunoprecipitation was used for input. The PCR products were analyzed by 2% agarose gel electrophoresis, and stained with ethyl bromide. The cell lines, specific antibodies, and targeted PCR fragments were indicated. The experiments were done three times and all produced similar results. The representative targeted PCR product images are depicted, and bars show mean ± SD. *: p < 0.01 as c-Myc compared to IgG.

The interactions between two E boxes of the *BRCA1 *promoter and c-Myc were investigated by an *in vivo *ChIP assay. The cross-linked protein-DNA complexes from cultured cells were immunoprecipitated with an anti-c-Myc antibody, and then DNA fragments were released from immunoprecipitates. The c-Myc-associated fragments were detected by semi-quantitative PCR analysis using primers designed to amplify the regions flanking each E box. Enrichment in fragments containing either E box X; or E box Y were found in immunoprecipitates treated with an anti-c-Myc antibody in comparison to the control IgG, as detected by semi-quantitative PCR using MCF-7 or MDA-MB-231 cells (Figure 4A, X and Y panels). In MCF 7 cells, there was nearly 4 or 5 fold increase in bound fragments containing E box X; or E box Y compared to the control IgG, respectively as determined by densitometry quantification. A similar enrichment was quantified in MDA-MB-231 cells; almost 6 or 4 fold more for E box X; or E box Y, respectively. There was no enrichment of PCR products in anti-c-Myc antibody-treated samples with primers designed to amplify a non E box region in BRCA1 promoter region, indicating this region was not associated with c-Myc (Figure 4A, non E box panel). To demonstrate the specificity of the E box X; or Y-c-Myc interaction, we repeated the CHIP assay with CCCTC binding factor (CTCF). CTCF binds to the *BRCA1 *promoter and plays a crucial role in maintaining the methylation-free state of the functional *BRCA1 *promoter region [33]. Although CTCF binds to the BRCA1 proximal region as we previous reported [33], at E box X; or Y there was no enrichment of targeted PCR products when using anti CTCF antibody comparing that of control IgG (Figure 4B), suggesting CTCF was not associated with these E boxes. These data strongly indicated that c-Myc associates with these E boxes in breast cancer cells.

### c-Myc activation of the BRCA1 promoter through E boxes

**Figure 5:**
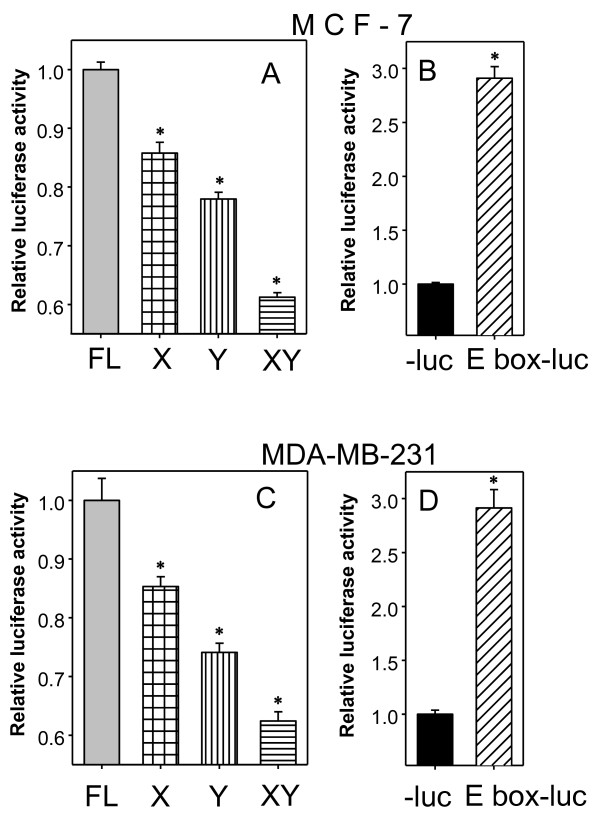
**E boxes in the *BRCA1 *promoter are required for *BRCA1 *activation by constitutively expressed c-Myc**. MCF-7 or MDA-MB-231 cells (A or C, respectively) were transiently co-transfected with Myc-GFP expression vector along with the *BRCA1 *promoter (pCYL42, FL) or its derivates with E box nucleotide substitutions (X, Y, or XY) as described in methods. Luciferase activities from cell lysates were determined 48 hours after transfection. The fold changes were normalized to the vector control and the full-length *BRCA1 *promoter activities set as 1. *: p < 0.01. The activation of a promoter/reporter construct containing six tandem E boxes (E box-luc) by constitutively expressed Myc-GFP was used as a control [B or D (MCF-7 or MDA-MB-231, respectively)]. Bars show mean ± SD. *: p < 0.01 as each indicated column compared to the FL column in A or C, and to -luc column in B or D.

To determine if c-Myc-mediated *BRCA1 *activation is dependent on E boxes X; and Y, we generated E box-specific nucleotide substitutions in the *BRCA1 *distal promoter. The pCYL42 promoter/reporter construct was used as a parental vector and the E box sequences CACTTG (E box X), or CACGTTG (E box Y) were mutated to CAAAAG, or AAAATTG, respectively, with nucleotide substitutions underlined. The *BRCA1 *promoter/reporter activities were monitored in the presence of constitutively expressed Myc-GFP. The activity of the *BRCA1 *promoters containing nucleotide substitutions in each E box was reduced compared to that of the wild-type in MCF-7 or MDA-MB-231 cells (Figure 5A or 5C, respectively). The promoter activity with double E box nucleotide substitutions was decreased to a greater degree (Figure 5A or 5C). To address the c-Myc dependent activation by Myc-GFP, we used an E box promoter/luciferase reporter construct, pMyc-TA-Luc, which contained six tandem E box consensus sequences inserted into pTA-Luc. The activity of the promoter containing E boxes (E box-Luc), induced by constitutively expressed Myc-GFP, was almost 3 times that of the promoter without an E box (-Luc) (Figure 5B or 5D, MCF-7 or MDA-MB-231, respectively). These observations and the results from the *in vivo *ChIP assay described above indicate that E boxes in the *BRCA1 *promoter region are sufficient for c-Myc dependent activation in cultured breast cancer cells.

### c-Myc dependent BRCA1 mediated DNA damage repair in breast cancer cells

**Figure 6:**
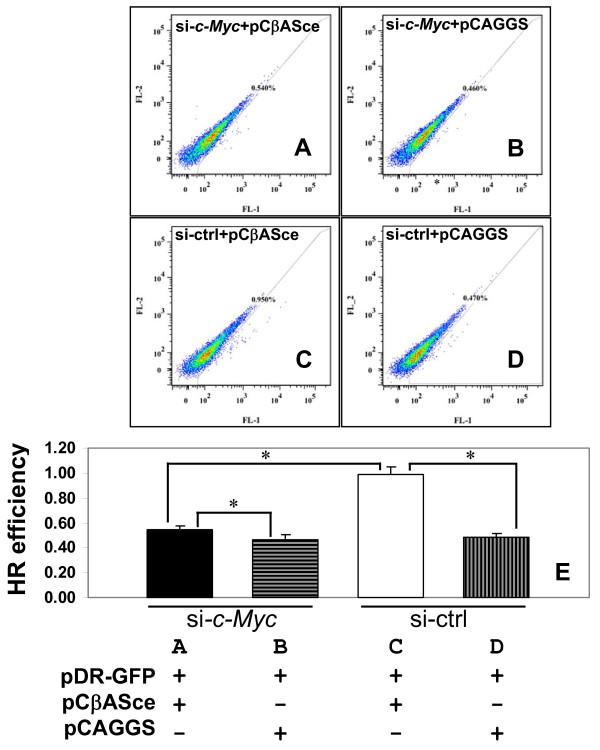
**Examination of HR activity using DR-GFP reporter**. MDA-MB-231 cells were treated with c-Myc siRNA or control siRNA, then the DR-GFP reporter plasmids with different combinations were co-transfected into cells as indicated in E. The percentage of GFP positive cell was determined by a flow cytometer using detector FL-1 (green) vs FL-2 (orange), the HR efficiency represented as the frequency of GFP-positive cell. The representative analysis for each treatment depicts as A, B, C, or D along with the percentage of GFP positive cells due to gene conversion as E. Bars show mean ± SD. *: p < 0.01 as compared with paired groups indicated.

BRCA1 is involved in chromosomal stability and it has been shown that BRCA1 deficient cells have impaired DNA damage repair [34]. After showing that reduced c-Myc expression led to decreased BRCA1 expression, we tested whether depletion of c-Myc could abrogate the BRCA1-mediated DNA repair function. We adopted a well characterized GFP based double strand break repair assay to monitor DNA repair activity. In this system, screening cells for GFP expression following homologous recombination was used for testing DNA repair activity. MDA-MB-231 cells were treated with c-Myc siRNA (si-Myc) to deplete c-Myc thus correlated with reduced BRCA1 expression, or control siRNA (si-ctrl) (Figure 1). Then the DR-GFP reporter system in various combinations was introduced into cells. 48 hours later the green fluorescence in the cells was monitored by a flow cytometer. The GFP positive cells were identified after the FL-1 (green fluorescence) vs FL-2 (orange fluorescence) ratio. The representative analyses for each condition of DR-GFP reporter system were depicted as A, B, C, or D and the summary of three triplicate experiments was shown in E. The control siRNA treated cells with intact BRCA1 exhibited considerable homologous recombination efficiency (HR efficiency) (Figure 6C, D, and 6E, si-ctrl). A significant reduction of HR efficiency was observed when c-Myc was depleted by c-Myc siRNA treatment compared to the cells with control siRNA (Figure 6A, C and, Figure 6E, black column vs blank column). A slightly higher DNA repair activity was evident between the cells in the presence of I-SceI and without I-SceI even both cells were treated with c-Myc siRNA (Figure 6A, B, and 6E, si-*c-Myc*), indicating cells still maintained HR activity at certain level even with reduced BRCA1. No significant difference in HR efficiency was found between c-Myc siRNA and control siRNA treated cells without I-SceI introduced DSBs (Figure 6B, D, and 6E, horizontal vs vertical lined column).

## Discussion

In the present report, we used a targeted identification strategy for characterizing regulatory DNA elements to show transcriptional activation of *BRCA1 *by c-Myc through c-Myc's interaction with two E boxes in the distal *BRCA1 *promoter region. Genome-wide analysis has shown that c-Myc could bind to the regulatory regions of up to 15% of all human genes, and these data also indicated that c-Myc-DNA interactions were not sufficient for promoter regulation in some cases [35,36]. Indeed, it has been shown that although c-Myc binds to the *CCL5 *gene promoter in both MCF-7 and MDA-MB-231 cells, c-Myc-dependent regulation of *CCL5 *was evident in MCF-7 but not in MDA-MB-231 cells. Consistently, gene expression profiling analysis has shown that the spectrum of genes regulated by c-Myc in these cell lines is intrinsic to each cell line [37]. While the mechanism of how c-Myc functions in such a cell context-dependent manner is not clear, possibilities include, but are not limited to, post-translational modifications of c-Myc. For example, phosphorylation and acethylation of c-Myc could affect its protein-DNA and protein-protein interactions, which would contribute to its selective binding of target gene promoters. Recently Benassi *et al*. showed that phosphorylation of S62 of c-Myc could increase its binding ability to the promoter region of the γ-glutamyl cysteine synthetase gene [38]. However, the conclusive connections between c-Myc-DNA binding abilities and c-Myc transcriptional activities in a given condition are still obscure, as in the case of the *CCL5 *gene. In this study, we identified two E boxes within the *BRCA1 *promoter region and provided evidence that these were necessary for c-Myc-dependent promoter activation in MCF-7 and MDA-MB-231 cells (Figure 1). Although the co-factors recruited by c-Myc to these sites were not elucidated in our present experiments, we speculate that it is highly likely that a c-Myc-containing transcription complex would play an important role in *BRCA1 *expression.

Our data showed that *BRCA1 *activation by c-Myc only increased promoter activities by 1.8 fold under the conditions used (Figure 2B), and disrupting c-Myc-responsive elements (two E boxes) by different combinations correlated with loss of promoter activity ranging from 30-40% (Figure 5A and 5C). This observation is consistent with a previous report that *BRCA1 *mRNA expression was detectable in *c-Myc *knockout *c-Myc^-/- ^*Rat1A cells, and expression of c-Myc by serum stimulation in the parent *c-Myc^+/+ ^*Rat1A cells slightly increased *BRCA1 *mRNA expression [27]. *BRCA1 *expression activated by c-Myc could be the final outcome of the interplay within multi-component transcriptional networks containing factors important for breast tumorigenesis. It has been shown that HIF-1α transcriptional machinery activated by hypoxia signaling pathway abrogates c-Myc activation of *BRCA1 *expression in colon cancer cells [39]. c-Myc activates the expression of *p53 *through an E box within the *p53 *promoter [40]. On the other hand, p53 represses *c-Myc *transcription by binding to the *c-Myc *promoter and recruiting the general repressor mSin3a; this repression is required for cell cycle arrest and differentiation but not apoptosis [41]. In addition, p53 up-regulates *ER-α *by increasing gene transcription [42]. c-Myc is up-regulated by activated ER-α and plays a critical role in enhancing estrogen-induced breast cell proliferation [43,44]. Although not directly mediated by the ER-α pathway, estrogen stimulation up-regulates *p53 *expression [45]. Thus, further studies to elucidate transcription factor occupancy on the *BRCA1 *promoter, including proximal and distal regions, in breast cancer cells with distinct molecular signatures would be critical to understanding the complex regulation of *BRCA1 *expression during tumor progression.

Accumulating evidence has demonstrated that BRCA1 is a major component of the DNA damage repair complex required for normal cellular processes. Depletion of BRCA1 has been associated with decreased DNA damage repair and increased chromosomal instability [46]. In order to maintain the basal level of DNA damage repair, BRCA1 may be required for c-Myc associated cell proliferation. In this report we show that c-Myc can activate *BRCA1 *expression in breast cancer cells, and depletion of c-Myc reduced BRCA1-dependent DNA repair. However, the complicated interaction between c-Myc and BRCA1 may be cell context dependent. In breast cancer patients, *c-Myc *amplification was found more often in patients with BRCA1 deficiency than in patients with normal BRCA1 [47], suggesting that c-Myc amplification could be linked to decreased BRCA1 but the mechanism is poorly understood. These data suggested that c-Myc overexpression and BRCA1 loss seemed highly correlated in a large portion of basal-like breast cancers. This could indicate that loss of BRCA1 with c-Myc overexpression might lead to the development of basal-like breast cancer. Interestingly, we have reported that reduction of BRCA1 expression could be due to *BRCA1 *promoter methylation [9]. c-Myc has been shown to recruit DNA methyl transferases such as DNMT3a to repress p21Cip1 gene expression [48]. It raises the questions of c-Myc's involvement in the DNA methylation complex in breast cancer cells; particularly if *BRCA1 *is a target. It is worth noting that aberrant expression of DNA methyl transferase or its alternative splicing forms have been detected in cancerous cells, and could affect the distribution of DNA methylation [49].

Although our data support a model in which *BRCA1 *expression is regulated by transcription factors, such as c-Myc, it has been shown that other mechanisms could also affect *BRCA1 *expression. Epigenetic regulation such as *BRCA1 *promoter methylation has been shown to account for BRCA1 loss in 10-31% of sporadic breast cancer tumors [9]. The accessibility of transcription factors to the methylated *BRCA1 *promoter is reduced, and consequently the expression of *BRCA1 *mRNA and BRCA1 protein is decreased [33]. On the other hand, nucleosome occupancy has been identified as an alternative means to regulate *BRCA1 *expression [50]. Taken together, chromatin structure, methylation status of the gene promoter and dynamic transcription factor recruitment may work together to affect BRCA1 expression in breast cancer cells. Newly identified regulation by means of microRNA could also play a role in *BRCA1 *expression at the translational level. Aberrant expression of a set of microRNAs have been found in breast tumor samples, and some of these microRNAs could target *BRCA1 *mRNA [51], thus possibly regulating *BRCA1 *expression.

## Conclusions

Previous data from our lab and others have shown that *BRCA1 *expression in breast cancer was regulated at epigenetic and transcriptional levels. The data presented here suggests c-Myc is a novel member of transcription activators for *BRCA1 *expression. This study showed that the E boxes in the distal *BRCA1 *promoter region contribute to *BRCA1 *gene activation, and this activation is dependent on c-Myc. Our data suggest a transcriptional link between c-Myc and BRCA1, which has consequences for BRCA1 dependent-DNA damage repair.

## Competing interests

The authors declare that they have no competing interests.

## Authors' contributions

YC and JX designed and conducted the experiments, and wrote the manuscript. SB and CC performed experiments, and DH participated in the experiments and conducted the statistical analysis. OIO conceived the study, participated in its design and coordination, and helped to draft the manuscript. All authors read and approved the final manuscript.

## Pre-publication history

The pre-publication history for this paper can be accessed here:

http://www.biomedcentral.com/1471-2407/11/246/prepub

## References

[B1] Antoniou AC Gayther SA Stratton JF Ponder BA Easton DF Risk models for familial ovarian and breast cancer Genet Epidemiol 2000 18 2 173 190 10.1002/(SICI)1098-2272(200002)18:2<173::AID-GEPI6>3.0.CO;2-R 10642429

[B2] Ford D Easton DF Bishop DT Narod SA Goldgar DE Risks of cancer in BRCA1-mutation carriers. Breast Cancer Linkage Consortium Lancet 1994 343 8899 692 695 10.1016/S0140-6736(94)91578-4 7907678

[B3] Ford D Easton DF Stratton M Narod S Goldgar D Devilee P Bishop DT Weber B Lenoir G Chang-Claude J Sobol H Teare MD Struewing J Arason A Scherneck S Peto J Rebbeck TR Tonin P Neuhausen S Barkardottir R Eyfjord J Lynch H Ponder BAJ Gayther SA Birch JM Lindblom A Stoppa-Lyonnet D Bignon Y Borg A Hamann U Genetic heterogeneity and penetrance analysis of the BRCA1 and BRCA2 genes in breast cancer families. The Breast Cancer Linkage Consortium Am J Hum Genet 1998 62 3 676 689 10.1086/301749 9497246 PMC1376944

[B4] Satagopan JM Offit K Foulkes W Robson ME Wacholder S Eng CM Karp SE Begg CB The lifetime risks of breast cancer in Ashkenazi Jewish carriers of BRCA1 and BRCA2 mutations Cancer Epidemiol Biomarkers Prev 2001 10 5 467 473 11352856

[B5] Struewing JP Hartge P Wacholder S Baker SM Berlin M McAdams M Timmerman MM Brody LC Tucker MA The risk of cancer associated with specific mutations of BRCA1 and BRCA2 among Ashkenazi Jews N Engl J Med 1997 336 20 1401 1408 10.1056/NEJM199705153362001 9145676

[B6] Rosen EM Fan S Pestell RG Goldberg ID BRCA1 gene in breast cancer J Cell Physiol 2003 196 1 19 41 10.1002/jcp.10257 12767038

[B7] Futreal PA Liu Q Shattuck-Eidens D Cochran C Harshman K Tavtigian S Bennett LM Haugen-Strano A Swensen J Miki Y Eddington K McClure M Frye C Weaver-Feldhaus J Ding W Gholami Z Söderkvist P Terry L Jhanwar S Berchuck A Iglehart JD Marks J Ballinger DG Barrett JC Skolnick MH Kamb A Wiseman RW BRCA1 mutations in primary breast and ovarian carcinomas Science 1994 266 5182 120 122 10.1126/science.7939630 7939630

[B8] Lancaster JM Wooster R Mangion J Phelan CM Cochran C Gumbs C Seal S Barfoot R Collins N Bignell G Patel S Hamoudi R Larsson C Wiseman RW Berchuck A Iglehart JD Marks JR Ashworth A Stratton MR Futreal PA BRCA2 mutations in primary breast and ovarian cancers Nat Genet 1996 13 2 238 240 10.1038/ng0696-238 8640235

[B9] Wei M Grushko TA Dignam J Hagos F Nanda R Sveen L Xu J Fackenthal J Tretiakova M Das S Olopade OI BRCA1 promoter methylation in sporadic breast cancer is associated with reduced BRCA1 copy number and chromosome 17 aneusomy Cancer Res 2005 65 23 10692 10699 10.1158/0008-5472.CAN-05-1277 16322213

[B10] Butcher DT Rodenhiser DI Epigenetic inactivation of BRCA1 is associated with aberrant expression of CTCF and DNA methyltransferase (DNMT3B) in some sporadic breast tumours Eur J Cancer 2007 43 1 210 219 10.1016/j.ejca.2006.09.002 17071074

[B11] Xu CF Brown MA Nicolai H Chambers JA Griffiths BL Solomon E Isolation and characterisation of the NBR2 gene which lies head to head with the human BRCA1 gene Hum Mol Genet 1997 6 7 1057 1062 10.1093/hmg/6.7.1057 9215675

[B12] Suen TC Goss PE Transcription of BRCA1 is dependent on the formation of a specific protein-DNA complex on the minimal BRCA1 Bi-directional promoter J Biol Chem 1999 274 44 31297 31304 10.1074/jbc.274.44.31297 10531328

[B13] Thakur S Croce CM Positive regulation of the BRCA1 promoter J Biol Chem 1999 274 13 8837 8843 10.1074/jbc.274.13.8837 10085126

[B14] Thakur S Nakamura T Calin G Russo A Tamburrino JF Shimizu M Baldassarre G Battista S Fusco A Wassell RP Dubois G Alder H Croce CM Regulation of BRCA1 transcription by specific single-stranded DNA binding factors Mol Cell Biol 2003 23 11 3774 3787 10.1128/MCB.23.11.3774-3787.2003 12748281 PMC155225

[B15] Atlas E Stramwasser M Whiskin K Mueller CR GA-binding protein alpha/beta is a critical regulator of the BRCA1 promoter Oncogene 2000 19 15 1933 1940 10.1038/sj.onc.1203516 10773883

[B16] Atlas E Stramwasser M Mueller CR A CREB site in the BRCA1 proximal promoter acts as a constitutive transcriptional element Oncogene 2001 20 48 7110 7114 10.1038/sj.onc.1204890 11704836

[B17] Mancini DN Rodenhiser DI Ainsworth PJ O'Malley FP Singh SM Xing W Archer TK CpG methylation within the 5' regulatory region of the BRCA1 gene is tumor specific and includes a putative CREB binding site Oncogene 1998 16 9 1161 1169 10.1038/sj.onc.1201630 9528858

[B18] Bindra RS Gibson SL Meng A Westermark U Jasin M Pierce AJ Bristow RG Classon MK Glazer PM Hypoxia-induced down-regulation of BRCA1 expression by E2Fs Cancer Res 2005 65 24 11597 11604 10.1158/0008-5472.CAN-05-2119 16357170

[B19] Bindra RS Glazer PM Basal repression of BRCA1 by multiple E2Fs and pocket proteins at adjacent E2F sites Cancer Biol Ther 2006 5 10 1400 1407 10.4161/cbt.5.10.3454 17106239

[B20] De Siervi A De Luca P Byun JS Di LJ Fufa T Haggerty CM Vazquez E Moiola C Longo DL Gardner K Transcriptional autoregulation by BRCA1 Cancer Res 70 2 532 542 10.1158/0008-5472.CAN-09-1477 PMC2952428 20068145

[B21] Rauch T Zhong X Pfeifer GP Xu X 53BP1 is a positive regulator of the BRCA1 promoter Cell Cycle 2005 4 8 1078 1083 15970701

[B22] Jeffy BD Hockings JK Kemp MQ Morgan SS Hager JA Beliakoff J Whitesell LJ Bowden GT Romagnolo DF An estrogen receptor-alpha/p300 complex activates the BRCA-1 promoter at an AP-1 site that binds Jun/Fos transcription factors: repressive effects of p53 on BRCA-1 transcription Neoplasia 2005 7 9 873 882 10.1593/neo.05256 16229810 PMC1501940

[B23] Hockings JK Thorne PA Kemp MQ Morgan SS Selmin O Romagnolo DF The ligand status of the aromatic hydrocarbon receptor modulates transcriptional activation of BRCA-1 promoter by estrogen Cancer Res 2006 66 4 2224 2232 10.1158/0008-5472.CAN-05-1619 16489025

[B24] Wardrop SL Brown MA Identification of two evolutionarily conserved and functional regulatory elements in intron 2 of the human BRCA1 gene Genomics 2005 86 3 316 328 10.1016/j.ygeno.2005.05.006 15990270

[B25] Dang CV O'Donnell KA Zeller KI Nguyen T Osthus RC Li F The c-Myc target gene network Semin Cancer Biol 2006 16 4 253 264 10.1016/j.semcancer.2006.07.014 16904903

[B26] Solomon DL Amati B Land H Distinct DNA binding preferences for the c-Myc/Max and Max/Max dimers Nucleic Acids Res 1993 21 23 5372 5376 10.1093/nar/21.23.5372 8265351 PMC310573

[B27] Menssen A Hermeking H Characterization of the c-MYC-regulated transcriptome by SAGE: identification and analysis of c-MYC target genes Proc Natl Acad Sci USA 2002 99 9 6274 6279 10.1073/pnas.082005599 11983916 PMC122939

[B28] Esteller M Epigenetic lesions causing genetic lesions in human cancer: promoter hypermethylation of DNA repair genes Eur J Cancer 2000 36 18 2294 2300 10.1016/S0959-8049(00)00303-8 11094302

[B29] Livak KJ Schmittgen TD Analysis of relative gene expression data using real-time quantitative PCR and the 2(-Delta Delta C(T)) Method Methods 2001 25 4 402 408 10.1006/meth.2001.1262 11846609

[B30] Weng L Zhu C Xu J Du W Critical role of active repression by E2F and Rb proteins in endoreplication during Drosophila development Embo J 2003 22 15 3865 3875 10.1093/emboj/cdg373 12881421 PMC169046

[B31] Pierce AJ Jasin M Measuring recombination proficiency in mouse embryonic stem cells Methods Mol Biol 2005 291 373 384 15502236 10.1385/1-59259-840-4:373

[B32] Sy SM Huen MS Zhu Y Chen J PALB2 regulates recombinational repair through chromatin association and oligomerization J Biol Chem 2009 284 27 18302 18310 10.1074/jbc.M109.016717 19423707 PMC2709360

[B33] Xu J Huo D Chen Y Nwachukwu C Collins C Rowell J Slamon DJ Olopade OI CpG island methylation affects accessibility of the proximal BRCA1 promoter to transcription factors Breast Cancer Res Treat 2009 10.1007/s10549-009-0422-1 PMC6697119 19466541

[B34] Scully R Ganesan S Vlasakova K Chen J Socolovsky M Livingston DM Genetic analysis of BRCA1 function in a defined tumor cell line Mol Cell 1999 4 6 1093 1099 10.1016/S1097-2765(00)80238-5 10635334

[B35] Fernandez PC Frank SR Wang L Schroeder M Liu S Greene J Cocito A Amati B Genomic targets of the human c-Myc protein Genes Dev 2003 17 9 1115 1129 10.1101/gad.1067003 12695333 PMC196049

[B36] Patel JH Loboda AP Showe MK Showe LC McMahon SB Analysis of genomic targets reveals complex functions of MYC Nat Rev Cancer 2004 4 7 562 568 10.1038/nrc1393 15229481

[B37] Cappellen D Schlange T Bauer M Maurer F Hynes NE Novel c-MYC target genes mediate differential effects on cell proliferation and migration EMBO Rep 2007 8 1 70 76 10.1038/sj.embor.7400849 17159920 PMC1796762

[B38] Benassi B Fanciulli M Fiorentino F Porrello A Chiorino G Loda M Zupi G Biroccio A c-Myc phosphorylation is required for cellular response to oxidative stress Mol Cell 2006 21 4 509 519 10.1016/j.molcel.2006.01.009 16483932

[B39] Koshiji M Kageyama Y Pete EA Horikawa I Barrett JC Huang LE HIF-1alpha induces cell cycle arrest by functionally counteracting Myc Embo J 2004 23 9 1949 1956 10.1038/sj.emboj.7600196 15071503 PMC404317

[B40] Reisman D Elkind NB Roy B Beamon J Rotter V c-Myc trans-activates the p53 promoter through a required downstream CACGTG motif Cell Growth Differ 1993 4 2 57 65 8494784

[B41] Ho JS Ma W Mao DY Benchimol S p53-Dependent transcriptional repression of c-myc is required for G1 cell cycle arrest Mol Cell Biol 2005 25 17 7423 7431 10.1128/MCB.25.17.7423-7431.2005 16107691 PMC1190302

[B42] Angeloni SV Martin MB Garcia-Morales P Castro-Galache MD Ferragut JA Saceda M Regulation of estrogen receptor-alpha expression by the tumor suppressor gene p53 in MCF-7 cells J Endocrinol 2004 180 3 497 504 10.1677/joe.0.1800497 15012604

[B43] Dubik D Dembinski TC Shiu RP Stimulation of c-myc oncogene expression associated with estrogen-induced proliferation of human breast cancer cells Cancer Res 1987 47 24 Pt 1 6517 6521 3677090

[B44] Park KJ Krishnan V O'Malley BW Yamamoto Y Gaynor RB Formation of an IKKalpha-dependent transcription complex is required for estrogen receptor-mediated gene activation Mol Cell 2005 18 1 71 82 10.1016/j.molcel.2005.03.006 15808510

[B45] Qin C Nguyen T Stewart J Samudio I Burghardt R Safe S Estrogen up-regulation of p53 gene expression in MCF-7 breast cancer cells is mediated by calmodulin kinase IV-dependent activation of a nuclear factor kappaB/CCAAT-binding transcription factor-1 complex Mol Endocrinol 2002 16 8 1793 1809 10.1210/me.2002-0006 12145335

[B46] Deng CX BRCA1: cell cycle checkpoint, genetic instability, DNA damage response and cancer evolution Nucleic Acids Res 2006 34 5 1416 1426 10.1093/nar/gkl010 16522651 PMC1390683

[B47] Grushko TA Dignam JJ Das S Blackwood AM Perou CM Ridderstrale KK Anderson KN Wei MJ Adams AJ Hagos FG Sveen L Lynch HT Weber BL Olopade OI MYC is amplified in BRCA1-associated breast cancers Clin Cancer Res 2004 10 2 499 507 10.1158/1078-0432.CCR-0976-03 14760071

[B48] Brenner C Deplus R Didelot C Loriot A Vire E De Smet C Gutierrez A Danovi D Bernard D Boon T Pelicci PG Amati B Kouzarides T de Launoit Y Di Croce L Fuks F Myc represses transcription through recruitment of DNA methyltransferase corepressor Embo J 2005 24 2 336 346 10.1038/sj.emboj.7600509 15616584 PMC545804

[B49] Ostler KR Davis EM Payne SL Gosalia BB Exposito-Cespedes J Le Beau MM Godley LA Cancer cells express aberrant DNMT3B transcripts encoding truncated proteins Oncogene 2007 26 38 5553 5563 10.1038/sj.onc.1210351 17353906 PMC2435620

[B50] Lin JC Jeong S Liang G Takai D Fatemi M Tsai YC Egger G Gal-Yam EN Jones PA Role of nucleosomal occupancy in the epigenetic silencing of the MLH1 CpG island Cancer Cell 2007 12 5 432 444 10.1016/j.ccr.2007.10.014 17996647 PMC4657456

[B51] Iorio MV Ferracin M Liu CG Veronese A Spizzo R Sabbioni S Magri E Pedriali M Fabbri M Campiglio M Menard S Palazzo JP Rosenberg A Musiani P Volinia S Nenci I Calin GA Querzoli P Negrini M Croce CM MicroRNA gene expression deregulation in human breast cancer Cancer Res 2005 65 16 7065 7070 10.1158/0008-5472.CAN-05-1783 16103053

